# Comparing the activPAL CREA and GHLA Algorithms for the Classification of Postures and Activity in Free-Living Children

**DOI:** 10.3390/ijerph192315962

**Published:** 2022-11-30

**Authors:** Duncan S. Buchan, Ukadike C. Ugbolue

**Affiliations:** Division of Sport and Exercise, School of Health and Life Sciences, University of the West of Scotland, Lanarkshire Campus, Scotland G72 0LH, UK

**Keywords:** agreement, equivalence, free-living, sedentary behaviour, accelerometry

## Abstract

The activPAL accelerometer has been used extensively in research to assess sedentary behaviour (SB) and physical activity (PA) outcomes. The aim of this study was to assess the comparability of PA and SB outcomes from two automated algorithms (CREA and GHLA) applied to the activPAL accelerometer. One hundred and twenty participants aged 8–12 years wore an activPAL accelerometer on their right thigh continuously for seven days on two occasions, providing valid data from 1058 days. The PALbatch software downloaded the data after applying the CREA and GHLA (latest) algorithms. The comparability of the algorithms were assessed using the mean absolute percent error (MAPE), intra-class correlation coefficients (ICC), and equivalence testing. Comparisons for daily wear time, primary lying, sitting and standing time, sedentary and stepping time, upright time, total number of steps, sit–stand transitions and stepping time ≤ 1 min revealed mainly small MAPE (≤2%), excellent ICCs (lower bound 95% CI ≥ 0.97), and equivalent outcomes. Time spent in sitting bouts > 60 min and stepping bouts > 5 min were not equivalent with the absolute zone needed to reach equivalence (≥7%). Comparable outcomes were provided for wear time and postural outcomes using the CREA or GHLA algorithms, but not for time spent in sitting bouts > 60 min and stepping bouts > 5 min.

## 1. Introduction

Leading a physically active lifestyle provides several health-related benefits in physical and mental health, cognitive function, and academic outcomes [1]. Yet in adolescents aged between 11–17 years, recent estimates suggest that 81% of adolescents are insufficiently active, with significant differences in prevalence estimates evident between genders and countries [2]. Alongside physical activity (PA), it is important to consider the role of sedentary behaviour in the health of children and adolescents. Sedentary behaviour (SB) can be defined “as any waking behaviour characterized by an energy expenditure ≤ 1.5 metabolic equivalents, while in a sitting, reclining, or lying position” [3]. In adults, excessive SB is associated with morbidity and all-cause mortality [4,5]. Although there is currently insufficient evidence available to establish whether there is a dose–response relationship between time spent sedentary and adverse health outcomes in children and adolescents, current understanding suggests that less time spent sedentary will be better for health outcomes [6]. This is reflected in current recommendations for children and adolescents offered by the World Health Organisation to limit the amount of time spent sedentary [1] and, internationally, through public health guidelines [7,8,9]. Given the importance given to reducing SB, accurate monitoring of this behaviour is essential.

The gold standard device for the objective measurement of SB is the thigh-worn activPAL (PAL Technologies Ltd., Glasgow, UK) [10]. Wearing the activPAL on the thigh enables users to differentiate between seated and upright postures as well as stationary and non-stationary activity, which can then be used to estimate time spent in sedentary and non-sedentary activities [11]. The validity of the activPAL device is well established, demonstrating a sensitivity of 96% to 98% for correctly identifying steps and posture against direct observation in laboratory settings [12,13]. Precise estimates of steps and posture from the activPAL has also been observed in free-living settings and has shown a sensitivity to reductions in sitting time [14]. Prior to late 2018, users only had the option of using activPAL’s proprietary VANE (standard) classification algorithm, which calculates time spent sitting, standing, and stepping as well as posture transitions. As the activPAL is often encouraged to be worn continuously throughout the monitoring period, the inability of the VANE algorithm to identify periods of non-wear time or time in bed can be problematic. Indeed, errors in the estimates of sedentary time can occur when time in bed is classified as sedentary time and when true sedentary time (i.e., lying down during the day) is misclassified as time in bed [15]. Equally, inflated estimates of sedentary time could occur when true non-wear time is not identified and misclassified as awake wear time. To overcome the limitations of the VANE algorithm, participants are requested to complete a log of non-wear times and in-out of bed times [11]. With this increased participant burden, however, logs are often incomplete, which raises the questions of how missing data are dealt with (if not described) and/or the inability to compare study findings if different methods are used.

With the full release of an enhanced “CREA” algorithm by the manufacturers of activPAL (PAL Technologies Ltd.) in mid 2019, users now have the ability to report on additional metrics to those provided by the VANE algorithm including non-wear time, time in bed, lying, cycling, and seated transport. The appeal for researchers being able to document time spent in different behaviours (i.e., sleep, PA, and SB) and explore their relationships with indicators of health is obvious [1,7]. Despite the appeal of activPAL’s CREA algorithm, limited studies have examined the equivalence of this algorithm against sleep logs or other available algorithms. In adults, Courtney et al. [16] reported that the CREA algorithm was equivalent to self-reported logs for time going to bed, but not for wake time, whereas Leister et al. [17] reported a lack of equivalence between self-reported logs and the CREA algorithm for sleep time (i.e., difference between bedtime and wake time). While these two studies focused on the accuracy of the time in and out of bed estimates of the CREA algorithm, others have compared the comparability of activity and posture outcomes provided by the VANE and CREA algorithms for adults [18]. In this study, the authors found equivalent estimates between the two activPAL algorithms for steps, activity score, stepping time, bouts of stepping, and upright time, but non-equivalent estimates for posture transitions and bouts of sitting.

Although these recent studies have extended our understanding of the comparability of the CREA algorithm with self-reported logs and other algorithms [16,17,18], they all involved adults. To the best of our knowledge, only one study has explored the agreement between self-reported diary and the CREA algorithm in children and adolescents [15]. Here, the authors reported the agreement between participant diaries and the CREA algorithm in a small sample of youths (n = 20) and found an overall accuracy of 90% for accurately classifying time out of bed. Despite also reporting non-significant differences between the CREA algorithm and participant diaries for several additional outcomes including out-of-bed time, total sedentary time, mean bout duration, number of breaks, etc., these findings included both adults and youth. Moreover, only a limited number of outcomes were reported in this study [15]. Given the recently proposed standardized Core Research Outcomes for Sedentary Behaviour research [19], it is important that future SB research begins to report these outcomes in order to enhance the accumulation of pooled evidence and opportunities for meta-analyses.

Continued advances in the assessment of SB and PA mean that software updates and new algorithms are routinely developed by manufacturers and are available for the user. In May 2022, a new updated “GHLA” algorithm was added to the activPAL software [20]. Other than stating that the GHLA algorithm is an updated version of the CREA algorithm with sensor calibration, no other information is provided. It is unclear therefore whether comparable outcomes are provided from the CREA and GHLA algorithms. To the best of our knowledge, no study has compared outputs from activPAL’s CREA and GHLA algorithms. Therefore, the purpose of this study is to compare activity and posture outcomes from the CREA and GHLA algorithms in free-living children and to establish which outcomes are equivalent.

## 2. Materials and Methods

The data used for the current study came from a previous study that explored the feasibility and acceptability of a classroom-based Active Breaks intervention [21]. Ethical approval for the study was received from the University of the West of Scotland with baseline measures undertaken in October 2018. Briefly, 146 child participants aged 8–12 years attending eight primary schools in North Lanarkshire, Scotland, volunteered to participate and provided baseline measures. Following the end of the intervention 6 weeks later, 117 participants completed the same baseline measures as described below.

Participants’ stature and mass were measured using a calibrated scale (Seca Digital Scales, Seca Ltd., Birmingham, UK) and stadiometer (Seca Stadiometer, Seca Ltd., Birmingham, UK), respectively, without shoes and in light clothing. Participants were asked to wear the activPAL Micro4 (PAL Technologies Ltd., Glasgow, UK; herein activPAL) accelerometer. The activPAL was placed into a nitril sleeve fitted to each participant on the anterior midline of their right thigh using hypoallergenic Hypafix (BSN Medical, Hull, UK) dressings. All participants were encouraged to wear the device at all times for 7 days. Prior to dissemination, each device was synchronized with Greenwich Mean Time and initialized using the manufacturers proprietary software (activPAL Professional, v7.2.28) to start data collection at 06:30 a.m. on the following day at 20 Hz. A valid day of wear time included ≥20 h of wear time, no data errors identified by the software, and 1440 min of recording time.

### 2.1. Data Processing

Upon the return of the activPAL devices, time- and date-stamped activPAL data files were immediately downloaded using the manufacturer proprietary software (activPAL Professional, v7.2.28) for later processing. Using the manufacturers proprietary software (PALbatch v8.11.1.36), the participant data were averaged across valid days using activPAL’s CREA algorithm (version 1.3) to provide the following outcomes, including wear time, valid wear time days, sedentary time, sitting time, stepping time, standing time, upright time, total number of steps, the number of sit–stand transitions, and primary lying. Primary lying is used by the CREA and GHLA (version 2.2) algorithms to estimate time spent sleeping. Alongside these measures, several additional outcomes were provided including time spent in sitting bouts longer than 30 and 60 min, and stepping time for the following durations of ≤1 min, >1 min to ≤5 min, >5 min to ≤10 min, >10 min to ≤20 min, and >20 min. The following outcomes were also provided from PALbatch when using the GHLA algorithm. To ensure consistency, the default of 10 s was used to classify the minimum non-upright and upright periods for both algorithms. Finally, outcomes provided by both algorithms were aligned for each participant.

### 2.2. Statistical Analysis

Participant data were averaged across valid days and separately at each data collection point. Once valid days were identified from the algorithms, the daily validation and daily summary outcome spreadsheets were downloaded to confirm the same valid days were identified by both algorithms. In addition, these spreadsheets were used to confirm no data errors were evident and that 1440 min of recording time was captured. Agreement between algorithms for each outcome was examined using mean percent error (MPE), mean absolute percent error (MAPE), intraclass correlation coefficients (ICC, two-way mixed effects, single measures, absolute agreement) with 95% confidence intervals (CI), equivalence tests and Bland–Altman plots as recommended [22]. MPE was provided to indicate the direction and magnitude of error at a group-level, whereas the MAPE provided an indicator of individual agreement by accounting for each participant’s error. To aid interpretations, a threshold of <5% for MPE was used to consider the practical relevance of the agreement between outcomes from each algorithm [23], whereas an MAPE of <3% denoted excellent agreement, as used elsewhere [18]. Values < 0.5, 0.5–0.75, 0.75–0.9, and >0.90 were indicative of poor, moderate, good, and excellent agreement, respectively, based on the lower bound 95%CI of the ICC estimate [24]. Finally, Bland–Altman plots were used to assess the agreement in outcomes between algorithms and to visualize the magnitude of differences [25].

The pairwise 95% equivalence tests were used to explore whether the 95% CI of the outcome mean from one algorithm fell within the proposed equivalence zone for each outcome [26]. Although applying a 10% equivalence zone is common in similar studies [27,28], such an approach can be problematic. For instance, the use of a 10% zone can be strict when values are highly variable and smaller but lax when values are very high and across a narrow range of values [29]. Therefore, the absolute equivalence zone needed to reach equivalence is provided alongside the relative equivalence zone presented as a proportion of the SD [30]. To aid interpretations, a strict threshold of 3%, indicative of excellent agreement between algorithms, was used to aid interpretations of the absolute equivalence zone [26]. All equivalence testing was undertaken twice. In the first instance, outcomes from the CREA algorithm were used as the reference method followed by outcomes provided by the GHLA algorithm. Statistical analyses were undertaken using IBM SPSS statistical software for Windows version 25 (IBM, Armonk, NY, USA), whereas equivalence testing was undertaken in Minitab (v17) with alpha set at 0.05.

## 3. Results

Of the 263 accelerometer data files that could have been provided, eight participants withdrew consent and four were removed by their teacher (n = 24), five participants failed to return their activPAL device, and one activPAL device suffered a battery malfunction shortly after distribution (n = 30). This left 233 accelerometer files from participants (n = 143) available for the subsequent analysis. Fifteen accelerometer files were further removed as they failed to provide ≥20 h of wear time for ≥1 day. This left 218 accelerometer files from 120 participants (68 girls; mean age: 10.4 ± 0.8 years) providing 1058 valid days, with an average of 4.9 valid days, to be processed in the subsequent analysis. The findings of the MPE, MAPE, and ICCs are provided in Table 1.

The findings from the MPE revealed that the group level differences for outcomes processed using the CREA and GHLA algorithms were all less than <5%. Findings from the MAPE revealed that individual level differences were mostly <3%, denoting excellent agreement in these outcomes between algorithms. Nonetheless, large MAPE values were evident for sitting bouts and stepping time > 5 to 10 min. Agreement from the ICCs were found to be excellent for all outcomes apart from stepping time > 5 to 10 min which demonstrated good agreement between the algorithms.

**Figure 1:**
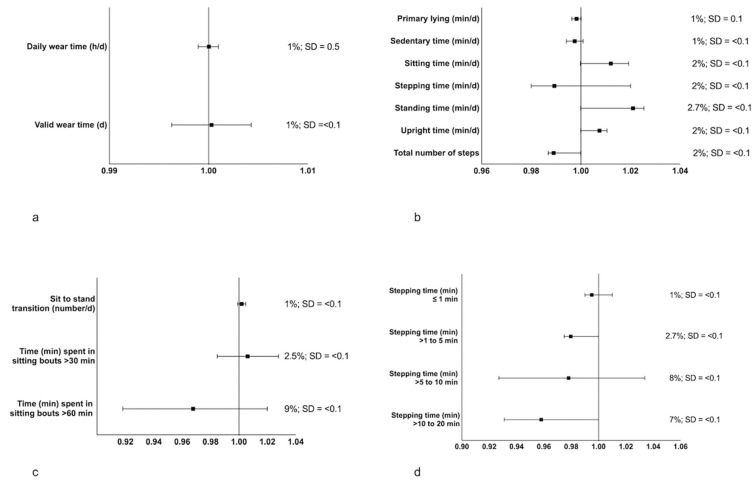
Equivalence of outcomes provided from the CREA and GHLA algorithms for (**a**) device wear time, (**b**) posture outcomes, (**c**) sitting bouts, and (**d**) stepping bouts. The solid squares represent the mean difference, whereas the horizontal lines show the 95% CI. To the right of each figure, the absolute zone needed to reach equivalence is provided as a %, alongside the zone necessary to achieve equivalence as a proportion of the SD. Outcomes from the CREA algorithm were used as the reference in all analyses and are presented here. All equivalence testing was repeated using the GHLA algorithm outcomes as the reference and provided identical results (not presented). An equivalence test not undertaken for stepping time > 20 min as only 13 cases provided values with the remaining comparisons all being zeros.

The findings from the equivalency analysis are displayed in Figure 1. The relative zone needed to reach equivalence for almost all outcomes was ≤0.1 SDs. Although the relative zone for total daily wear-time was 0.5 SDs, this was likely a consequence of the very small SD for this outcome. The absolute zone needed to reach equivalence was in the main ≤ 3%, apart from time spent in sitting bouts > 60 min, stepping time > 5 to 10 min, and stepping time > 10 to 20 min, which demonstrated absolute equivalence zones ranging from 7% to 9%.

**Figure 2:**
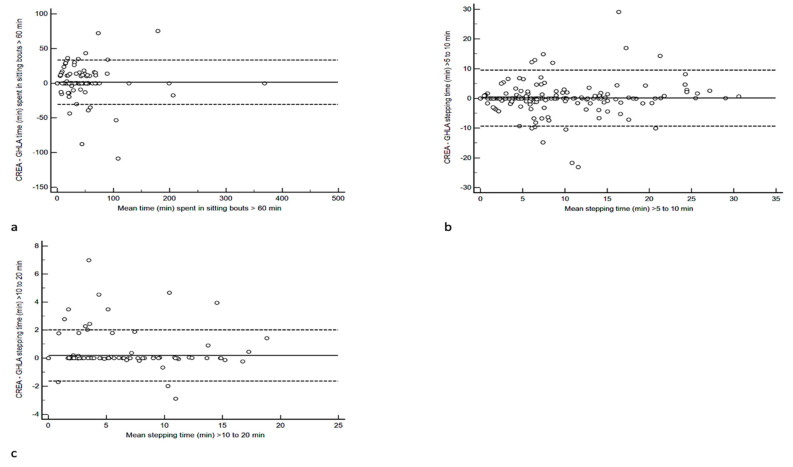
Bland–Altman plots comparing the outcomes provided from the CREA and GHLA algorithms for (**a**) time (min) spent in sitting bouts > 60 min, (**b**) stepping time (min) > 5 to 10 min, and (**c**) stepping time (min) > 10 to 20 min.

To examine the differences in these outcomes in more detail, Bland–Altman plots were undertaken with the findings displayed in Figure 2. The mean bias between the CREA and GHLA algorithms for time spent in sitting bouts > 60 min was 1.3 min with limits of agreement (LoA) of −31 to 33 min. The mean bias for stepping time > 5 to 10 min was 0.1 min with LoA of −9.4 to 9.5 min, whereas the mean bias for stepping time > 10 to 20 min was 0.2 min with LoA of −1.6 to 2.0 min.

## 4. Discussion

The aim of this study was to compare the comparability of outcomes collected from activPAL devices using PAL Technologies CREA and GHLA algorithms. Excellent agreement and equivalence of outcomes were evident for measures of daily wear time and posture outcomes. At a group level, the differences for daily wear time, valid days, and most postural outcomes (primary lying, sedentary time, sitting time, stepping time, upright time, and total number of steps) were ≤1%. These findings suggest that near identical values are provided for these outcomes when calculated by the CREA and GHLA algorithms at the group level. For the number of sit–stand transitions, time spent in sitting bouts > 30 min, stepping time ≤ 1 min, >1 to 5 min, and >20 min, near identical values were provided by the CREA and GHLA algorithms, demonstrating excellent comparability. Yet, the high absolute zone needed to reach equivalence for time spent in sitting bouts > 60 min and stepping time > 5 to 10 min and 10 to 20 min suggest caution is warranted if looking to compare these outcomes when processed using different algorithms. These findings suggest that device wear time and postural outcomes calculated using the CREA and GHLA algorithms are comparable and may facilitate study comparisons. Conversely, caution is advised when comparing time spent in sitting bouts > 60 min and bouts of stepping time > 5 min across studies if calculated using different algorithms.

To the best of our knowledge, this is the first study to compare outcomes from the CREA and GHLA algorithms. As such, it is difficult to draw comparisons between our findings and those previously published. In this study, poor agreement and a lack of equivalence was evident between algorithms for time spent in sitting bouts > 60 min. This is similar to a previous study, albeit involving older adults, that compared outcomes from the VANE and CREA algorithms [18]. Here Montoye et al., (2022) reported an MPE of 531% and a MAPE of 141% for time spent in sitting bouts > 60 min with the VANE algorithm providing the larger values. Similar MPE and MAPE values were also observed when comparing overall sitting time. The lack of comparability noted by the authors for overall sitting time is unsurprising given that the VANE algorithm combines sitting, lying down, and non-wear time into a single metric, whereas the CREA algorithm provides these as separate outcomes. In an attempt to prove a fairer comparison for sitting time, an adjusted sitting time outcome was calculated for the CREA algorithm by summing the overall sitting + primary lying + secondary lying + non-wear. The adjusted sitting time outcome subsequently demonstrated good agreement and equivalence between algorithms. Unfortunately, similar adjustments were not reported for time spent in sitting bouts > 60 min. In our study, overall sitting time demonstrated good comparability between the CREA and GHLA algorithms, which suggests that both algorithms calculate this outcome in a similar way.

An outcome that can affect time sitting is the number of transitions from a sedentary to upright posture, and vice versa. In the study by Montoye et al. (2022), poor comparability was evident between the number of sedentary to upright transitions (and vice versa) between the VANE and CREA algorithms. With the cause of differences in nearly all participant files being a result of the VANE algorithm identifying more transitions than the GHLA algorithm. In this study, excellent comparability was evident in the number of sit–stand transitions identified by the algorithms, with near identical values provided. Similar findings were also evident for the number of stand–sit transitions (data not reported). Therefore, our findings suggest that the poor comparability of time spent in sitting bouts > 60 min was not a consequence of how the algorithms calculated posture transitions or overall sitting time. When exploring the data in more detail, 17 files had to be removed from the MPE and MAPE analysis as one algorithm provided data (CREA = 12; GHLA = 5) when the other algorithm did not. From the outliers identified within the B&A plot (Figure 2a), most of the outliers were a result of the GHLA algorithm providing a higher value than the CREA algorithm, with differences ranging from 109%–202%.

When using the batch processing software PALbatch, both the CREA and GHLA algorithms provided details of accelerometer alignment with a value of 100%, indicative of the device being 100% aligned, whereas 50% alignment is indicative of a 45-degree angle offset [31]. None of the outlier files were perfectly aligned with differences ranging from 0% to 6%, and in all cases the alignment from CREA was greater than that from GHLA. For the three outliers that had the greatest differences in alignment (4%, 5%, and 6%), differences in time spent in sitting bouts > 60 min were 202%, 109%, and 158%, respectively. It is worth noting, nonetheless, that other outliers that had differences in alignment of 0% and 2% had differences of 113% and 177%, respectively. In the absence of an agreed consensus on how to deal with files that are not perfectly aligned, we examined the effect of excluding those files (n = 24) that displayed ≥3% differences in alignment. We found that MPE reduced slightly to <3%, whereas MAPE was reduced to 8.2%. Similar positive effects were also seen on the absolute zone needed to reach equivalence. Future work should therefore consider the impact of device alignment on outcomes, and whether there is a need to establish a consensus on the degree of alignment needed to warrant the inclusion of files in subsequent analyses. Finally, the alignment of all files from CREA was slightly greater than that from GHLA (95.8% vs. 94.6%, respectively) with both MPE and MAPE below 1.5%.

We also observed poor comparability for stepping time in bouts that were longer than 5 min. Despite excellent agreement at the group level for stepping time in bouts > 5 to 10 min, the MAPE was high at 142%. This is likely a consequence of the occasional outlier as seen from the B&A plot in Figure 2b, which resulted in wide SDs. Furthermore, time spent stepping in bouts > 5 to 10 min was generally low and on average less than 8 min, regardless of the algorithm, with no participant spending more than 31 min in stepping bouts > 5 to 10 min. There were also a considerable number of files that had to be removed from the analysis for this outcome (n = 45), as on occasion, one algorithm provided data (CREA = 24; GHLA = 21) and the other did not. Absolute differences between algorithms can thus appear substantial, when in fact the differences are only a matter of minutes. When examining those files that demonstrated a MAPE difference greater than 100% (n = 31, range 104% to 2738%), GHLA provided higher values than CREA for all comparisons. With the difference in alignment between these 31 files being less than 0.5%, these findings suggest that there are differences in how these two algorithms calculate stepping time longer than 5 min. Although we found excellent agreement for the number of sit–stand transitions (and vice versa) and overall stepping time, our findings do appear to suggest that algorithms calculate stepping bouts differently.

The importance of breaking up prolonged bouts of SB for health are reflected in current recommendations for children and adolescents that recognize the benefits of reducing the quantity of SB [1,7,8,9]. As our understanding of the most effective means of breaking up SB to induce health benefits evolves [32,33], it is important that valid measures of this behaviour are available. As this study used previously collected data however, we were unable to establish which algorithm is more accurate for measuring time spent in sitting and stepping bouts. Further work should therefore explore the validity of CREA and GHLA and establish which is more accurate for identifying time spent in sitting and stepping bouts across different durations.

The strengths of this study include the large sample of children that provided, on average, five free-living days of valid wear time to be analysed. The use of several standardized approaches to explore the comparability of outcomes provided detailed comparisons, allowing future studies to compare their findings to ours. Furthermore, being able to download data from one device removed the need for additional devices and concerns over different device placements when comparing outcomes. There were some limitations to the study, including the lack of criterion measure to establish which algorithm was the most accurate for measuring outcomes where poor agreement was evident. Finally, the sample was recruited from one geographical area and comprised of children of a narrow age range, which may limit the generalizability of our findings to younger children and adolescents.

## 5. Conclusions

In conclusion, our findings suggest that comparable findings were evident for outcomes reporting daily wear time, posture, transitions between sedentary and upright postures, as well as time spent in sitting bouts > 30 min and stepping time in bouts lasting less than 5 min when calculated using PAL Technologies CREA and GHLA algorithms. These findings suggest that these outcomes may be compared across studies that have used either of these algorithms. Caution is advised if looking to compare time spent in sitting bouts > 60 min and stepping time > 5 to 10 min and 10 to 20 min if calculated using the CREA and GHLA algorithms. Future work should thus provide details of the algorithm used to calculate the outcomes from the activPAL device to facilitate study comparisons.

## Figures and Tables

**Table 1:** Comparison of outcomes provided from the CREA and GHLA algorithms.

Domain & Reporting Statistic	Outcome	CREA Algorithm	GHLA Algorithm	MPE ± SD	MAPE ± SD	ICC (95% CI)
Device Wear Time	Daily wear time (h/d)	23.9 ± 0.5	23.9 ± 0.5	−0.01 ± 0.37	0.02 ± 0.20	0.99 (0.99, 0.99)
	Valid wear time days	4.9 ± 1.5	4.9 ± 1.5	−0.09 ± 0.33	0.40 ± 3.45	0.99 (0.99, 0.99)
Posture Outcomes	Primary lying (min/d)	606.6 ± 56.1	605.6 ± 57.1	0.16 ± 1.86	0.19 ± 1.50	0.99 (0.99, 0.99)
	Sedentary time (min/d)	515.6 ± 73.1	514.9 ± 77.7	0.13 ± 5.97	1.35 ± 2.57	0.99 (0.99, 0.99)
	Sitting time (min/d)	347.3 ± 77.6	351.3 ± 78.8	−1.13 ± 1.50	3.76 ± 6.14	0.98 (0.97, 0.98)
	Stepping time (min/d)	132.7 ± 33.1	131.4 ± 33.1	1.00 ± 0.16	1.28 ± 1.87	0.99 (0.99, 0.99)
	Standing time (min/d)	175.6 ± 50.1	179.8 ± 51.2	−2.22 ± 4.24	3.43 ± 2.94	0.98 (0.97, 0.99)
	Upright time (min/d)	308.3 ± 69.8	311.1 ± 71.9	−0.86 ± 2.94	1.96 ± 2.02	0.99 (0.98, 0.99)
	Total number of steps	10,587.2 ± 2780.7	10,481.6 ± 2784.9	1.01 ± 0.17	1.25 ± 2.00	0.99 (0.99, 0.99)
Sitting bouts	Sit–stand transitions (number/d)	92 ± 22	92 ± 22	−0.22 ± 0.07	1.67 ± 2.01	0.99 (0.99, 0.99)
	Time (min) spent in sitting bouts > 30 min	92.9 ± 66.3	92.8 ± 66.3	−0.70 ± 3.22	7.92 ± 16.92	0.94 (0.93, 0.96)
	Time (min) spent in sitting bouts > 60 min	26.1 ± 44.3	25.7 ± 45.4	3.21 ± 0.96 *	10.32 ± 29.37	0.96 (0.95, 0.97)
Stepping bouts	Stepping time (min) ≤ 1 min	82.6 ± 19.6	82.2 ± 19.6	0.41 ± 0.63	1.23 ± 1.85	0.99 (0.99, 0.99)
	Stepping time (min) > 1 to 5 min	39.1 ± 14.8	38.3 ± 15.1	1.61 ± 1.22	3.46 ± 4.57	0.99 (0.99, 0.99)
	Stepping time (min) > 5 to 10 min	7.8 ± 7.3	7.8 ± 6.9	2.18 ± 8.76 ^†^	142.3 ± 287.1	0.78 (0.72, 0.83)
	Stepping time (min) > 10 to 20 min	3.0 ± 4.6	2.8 ± 4.5	1.98 ± 4.50 ^‡^	2.58 ± 9.92	0.98 (0.97, 0.98)
	Stepping time (min) > 20 min	0.3 ± 1.4	0.3 ± 1.4	0.26 ± 0.16	0.02 ± 0.31	0.97 (0.96, 0.98)

MPE = mean percent error; MAPE = mean absolute percent error; ICC = interclass correlation coefficient. * n = 201 as 17 files were removed as one algorithm provided data (CREA = 12; GHLA = 5) and the other did not. ^†^ n = 173 as 45 files were removed as one algorithm provided data (CREA = 24; GHLA = 21) and the other did not. ^‡^ n = 213 as 5 files were removed as one algorithm provided data (CREA = 4; GHLA = 1) and the other did not.

## Data Availability

The data presented in this study are available upon reasonable request from the corresponding author.
